# Tetraoctylammonium, a Long Chain Quaternary Ammonium Blocker, Promotes a Noncollapsed, Resting-Like Inactivated State in KcsA

**DOI:** 10.3390/ijms22020490

**Published:** 2021-01-06

**Authors:** Ana Marcela Giudici, Clara Díaz-García, Maria Lourdes Renart, Ana Coutinho, Manuel Prieto, José M. González-Ros, José Antonio Poveda

**Affiliations:** 1 Instituto de Investigación, Desarrollo e Innovación en Biotecnología Sanitaria de Elche (IDiBE), Instituto de Biología Molecular y Celular (IBMC), Universidad Miguel Hernández, E-03202 Elche, Spain; marcela@umh.es (A.M.G.); lrenart@umh.es (M.L.R.); 2 Institute for Bioengineering and Bioscience (IBB), Instituto Superior Técnico, Universidade de Lisboa, 1049-001 Lisboa, Portugal; clara.dg93@gmail.com (C.D.-G.); ana.coutinho@tecnico.ulisboa.pt (A.C.); manuel.prieto@tecnico.ulisboa.pt (M.P.); 3 Departamento de Química e Bioquímica, Faculty of Sciences, Universidade de Lisboa, 1749-016 Lisboa, Portugal

**Keywords:** potassium channels, tetraalkylammonium salts, protein thermal stability, homo-FRET, C-type inactivation, binding affinity, selectivity filter conformation, steady-state and time-resolved fluorescence anisotropy

## Abstract

Alkylammonium salts have been used extensively to study the structure and function of potassium channels. Here, we use the hydrophobic tetraoctylammonium (TOA^+^) to shed light on the structure of the inactivated state of KcsA, a tetrameric prokaryotic potassium channel that serves as a model to its homologous eukaryotic counterparts. By the combined use of a thermal denaturation assay and the analysis of homo-Förster resonance energy transfer in a mutant channel containing a single tryptophan (W67) per subunit, we found that TOA^+^ binds the channel cavity with high affinity, either with the inner gate open or closed. Moreover, TOA^+^ bound at the cavity allosterically shifts the equilibrium of the channel’s selectivity filter conformation from conductive to an inactivated-like form. The inactivated TOA^+^–KcsA complex exhibits a loss in the affinity towards permeant K^+^ at pH 7.0, when the channel is in its closed state, but maintains the two sets of K^+^ binding sites and the W67–W67 intersubunit distances characteristic of the selectivity filter in the channel resting state. Thus, the TOA^+^–bound state differs clearly from the collapsed channel state described by X-ray crystallography and claimed to represent the inactivated form of KcsA.

## 1. Introduction

Quaternary ammonium salts (QAs) have long been used as channel blockers in the characterization of K^+^ channels [1,2]. Initial studies were performed on the giant squid axon and *D. melanogaster* Shaker channels [3,4,5,6,7,8,9], and it has already been suggested that QAs bind to the conduction pore, hence impeding the K^+^ current. Nonetheless, the length of the alkyl chains and the hydrophobicity of the different QAs determine differences in their molecular mechanisms of blockade. Thus, while the shorter-chain QAs seem to block the channel by simply obstructing the conduction pathway, the longer-chain, more hydrophobic derivatives are also believed to induce a slow channel inactivation process [6].

**Scheme 1:**
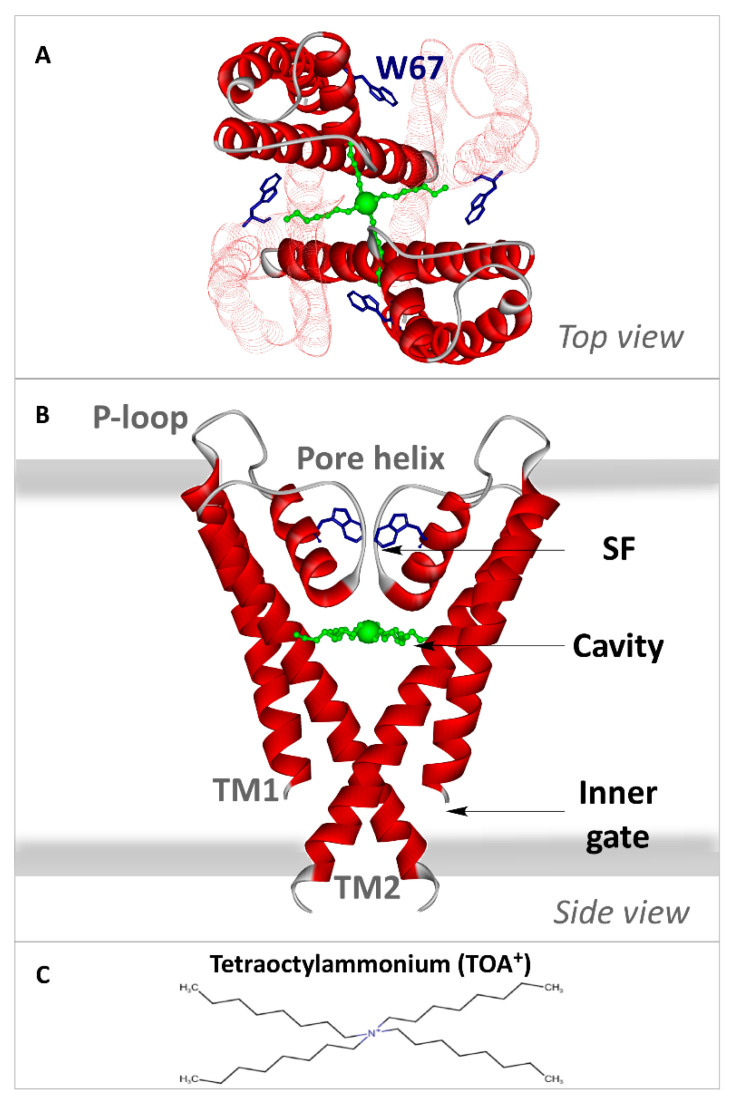
Transmembrane portion of KcsA. Crystallographic structure of the C-terminal truncated KcsA in the closed state with TOA^+^ (green balls and sticks, with a larger ball representing the quaternary nitrogen) bound at the cavity (PDB: 2W0F). In the top view (panel (**A**)), two of the four subunits appear faintly drawn to facilitate the observation. The four W67 residues are depicted as blue sticks. In the side view (panel (**B**)), only two of the four monomers have been drawn as a solid red ribbon for the sake of clarity. Each monomer consists of two transmembrane helices (TM1 and TM2) connected by the P-loop region, a short, tilted pore helix, and the selectivity filter (SF). The thick grey lines indicate the membrane limits. Both views illustrate how the TOA^+^ alkyl chains traverse the TM2 helix. Panel (**C**) represents the tetraoctylammonium ion in perspective.

The elucidation of molecular details on the interaction between QAs and K^+^ channels have advanced significantly with the resolution of the X-ray structures of QAs bound to KcsA, a prokaryotic K^+^ channel from *Streptomyces lividans*. This channel is a homotetrameric membrane protein, where each monomer includes two α-helical transmembrane segments (TM1 and TM2) and N- and C-terminal cytoplasmic ends (Scheme 1). The four C-terminal ends are arranged as a helical bundle, with a conformation that is sensitive to pH, acting as a channel gate (inner gate) [10]. On the other hand, the channel pore includes an aqueous cavity, a short tilted helix, and a selectivity filter (SF) with the sequence TVGYG, homologous to that of the eukaryotic K^+^ channels [11], that constitutes a second channel gate (outer gate). The backbone carbonyls of the SF residues conform to four K^+^ binding sites (sites S1–S4, from the extracellular to the intracellular side) [11,12], which can adopt different conformations at high or low K^+^ concentrations [12,13,14,15,16,17,18,19,20,21,22]. The conformation at a low K^+^ concentration shows no ions at the center of the SF (sites S2 and S3), thus adopting a “collapsed” structure that impedes ion flow through it. K^+^ binds to S1 and S4 in this conformation, with an average occupancy of just one ion distributed between those two sites. However, at high K^+^ concentrations, a conformational change is induced by a second K^+^ entering the filter, with a final average occupancy of two K^+^ ions per channel, either at the S1–S3 or the S2–S4 positions, thus enabling ion conduction [12,13,17,22]. The nonpermeant Na^+^, on the other hand, does not induce such a conformational change and shows an average occupancy of one ion per channel at the S1 and S4 sites [23].

In terms of functional activity, KcsA undergoes a cycle that includes four main different states, which reveals the concerted action of the two channel gates. At neutral pH and in the presence of K^+^, the channel is in a closed/conductive resting state, whereby the cytoplasmic inner gate impedes ion flow, while the SF (the outer gate) displays a conductive form. At acidic pH, the inner gate opens, allowing ion flow in this open/conductive state and making KcsA a proton-activated channel [24,25,26]. However, this is not a stable state, and the outer gate evolves to a conformation reminiscent of C-type inactivation in eukaryotic K^+^ channels [27,28,29], which impedes ion flow in this open/inactivated form [27,28,30]. Such an inactivation process is modulated by a network of interactions that includes the so-called inactivation triad, i.e., residues E71, D80, and W67 from each subunit [17]. The cycle is completed when the pH returns to neutrality, which closes the inner gate, causing the transient closed/inactivated state to evolve to the initial closed/conductive resting state [31].

The crystallographic studies on KcsA complexed to different QAs reveal that the QA binding site for the hydrophilic ammonium head group is located in the internal water-filled cavity of the channel, directly underneath the innermost cation binding site (S4) of the SF (see Scheme 1). The QAs are further stabilized in the cavity through the insertion of their alkyl chains of varying lengths into the hydrophobic channel wall so that this hydrophobic component becomes an important source of binding stability. Thus, hydrophobic compounds such as TBA^+^ (tetrabutylammonium), THA^+^ (tetrahexylammonium) and TOA^+^ (tetrabutylammonium) bind to KcsA with very high affinity (nM range) [32,33]. In the particular case of TOA^+^–KcsA complexes, the X-ray structure also reveals that the channel SF is in a collapsed conformation at pH 7.0 and high K^+^ concentrations, i.e., the S2 and S3 K^+^ binding sites are absent, similar to that previously seen in the collapsed X-ray structure of the channel alone in the presence of very low K^+^ concentrations. This led those authors to conclude that such a collapsed structure is an inactivated state induced by the binding of TOA^+^. However, those X-ray studies used an L90C KcsA mutant without the C-terminal domain, in which the additional presence of a Fab fragment bound to the extracellular channel loop is suspected to restrict the conformational plasticity of the SF [17,34]. Indeed, previous studies from our laboratory on several inactivated models of KcsA, some of which were also predicted as collapsed from X-ray studies, have shown that the stack of K^+^ binding sites in the inactivated filters remains accessible to cations, as in the resting channel. Therefore, rather than being collapsed, the inactivated SF seems “resting-like” [35]. In apparent agreement with our observations, other authors found only modest conformational changes in the G77 residue during inactivation compared to the resting state [36], further supporting the tenet that the SF remains “resting-like” upon inactivation, with all four K^+^ binding sites accessible to cations. Our goal in this paper is to contribute to the elucidation of this controversy on the conformation of the SF in the inactivated channel state by studying in detail the effects of TOA^+^ binding to the KcsA channel, which, up to now, was believed to result in an inactivated channel with a collapsed SF [33].

## 2. Results

### 2.1. TOA^+^ Binds to Wild-Type KcsA with High Affinity and Diminishes the Thermal Stability of the Channel

**Figure 1:**
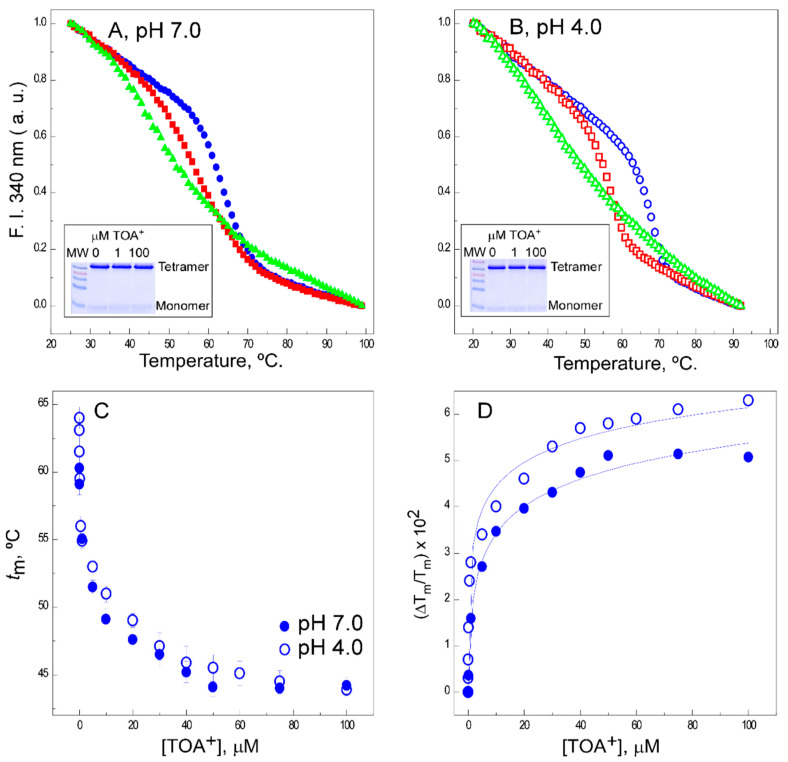
Effects of tetraoctylammonium (TOA^+^) on the thermal denaturation of wild-type (WT) KcsA. The intrinsic fluorescence at pH 7.0 (panel (**A**)) and pH 4.0 (panel (**B**)) and in the absence of TOA^+^ (full and empty circles, respectively) or upon the addition of 1 µM (full and empty squares, respectively) or 100 µM (full and empty triangles, respectively) of TOA^+^. The insets show SDS-PAGE analysis of the different samples to illustrate the integrity of tetrameric WT KcsA at room temperature. Panel (**C**) shows the dependence of the midpoint temperature of the protein denaturation process (*t*_m_) of WT KcsA with increasing concentrations of TOA^+^, either at pH 7.0 (●) or pH 4.0 (○). The results are the average *t*_m_ (in Celsius) ± S.D. from three independent titrations. Panel (**D**) illustrates the fitting of Equation (1) to the experimental data from Panel (**C**) (see Methods). The apparent dissociation constants for the TOA^+^-channel complexes and their 95% confidence intervals estimated at pH 7.0 and pH 4.0 were 1.8 (0.54–6.02) × 10^−7^ M and 3.6 (1.89–6.98) × 10^−8^ M, respectively. The observed differences between such dissociation constant (K_D_) values were not statistically significant.

An assay based on the thermal denaturation of the KcsA protein was previously developed to study the binding of different channel ligands to KcsA, including permeant or nonpermeant cations, membrane lipids, and others [35,37,38,39,40]. Figure 1A,B shows examples of thermal denaturation curves to illustrate that the addition of TOA^+^ at micromolar concentrations to the detergent-solubilized channel leads to a concentration-dependent decrease in the thermal stability of the wild-type protein, both at pH 7.0 and pH 4.0. Because of the destabilizing effects of TOA^+^ on KcsA, these experiments were always conducted at a constant 10-mM concentration of Na^+^ to ensure that the tetrameric channel does not dissociate into monomers at the different TOA^+^ concentrations tested (Figure 1A,B insets).

The midpoint temperatures (*t*_m_s) from the thermal denaturation curves at different TOA^+^ concentrations were used to build titration binding curves, such as those shown in Figure 1C. The fitting of such curves to a simple two-state binding equilibrium (see Methods) allows us to estimate the apparent dissociation constants (K_D_s) for the binding of TOA^+^ to WT KcsA, which were in the 10^−7^ and 10^−8^ M range at pH 7.0 and pH 4.0, respectively (Figure 1D). These indicate that TOA^+^ has a very high affinity for binding to the channel under either experimental condition. This is in contrast with previous observations on the binding to KcsA of tetrabutylammonium (TBA^+^), a shorter acyl chain QA, for which the binding affinity at pH 7.0 (K_D_ ~5 × 10^−9^ M) is similar to that reported here for TOA^+^ but drops five orders of magnitude at pH 4.0 (K_D_~3.5 × 10^−4^ M) [35]. Such a dramatic difference in binding affinities could be rationalized based on the crystallographic information on the binding site for these compounds in the channel protein [12,32,33,41] As indicated in our introduction, the channel-bound QAs are further stabilized at the cavity through the interaction of their alkyl chains with the hydrophobic channel wall. In fact, the long alkyl chains of TOA^+^ completely traverse the channel protein wall [33], so that hydrophobic interactions become particularly important to stabilize the TOA^+^–KcsA complex.

### 2.2. The Presence of TOA^+^ in the Cavity Diminishes the Affinity of WT KcsA to Bind K^+^ in the Closed Channel State

**Figure 2:**
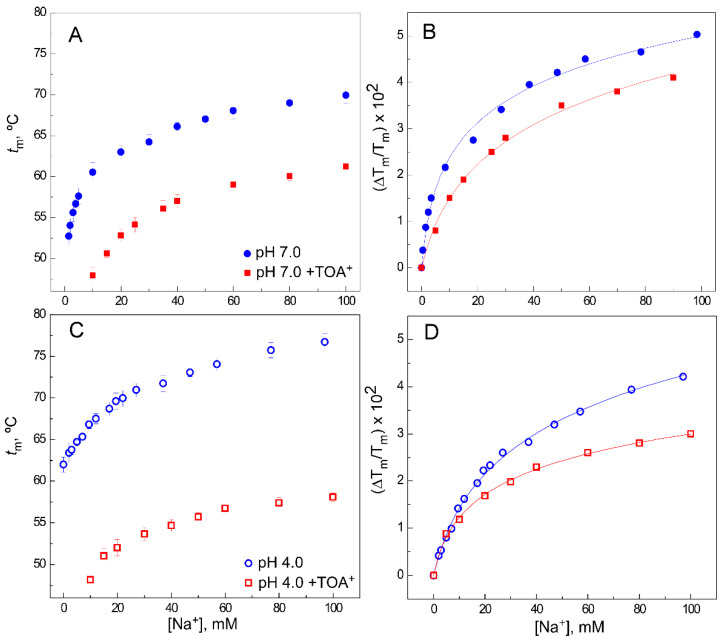
Effect of TOA^+^ on Na^+^ binding to WT KcsA. Panels (**A**,**C**) illustrate Na^+^ binding to the WT KcsA channel at pH 7.0 (panel (**A**)) or pH 4.0 (panel (**C**)), in the absence (circles) and presence (squares) of 100 µM TOA^+^, monitored through the Na^+^-concentration-dependence of the midpoint temperature of the protein denaturation process (*t*_m_). Each experimental point is the average *t*_m_ (in Celsius) ± S.D. (*n* = 3). Panels (**B**,**D**) show the fitting of Equation (1) to the experimental data from panels (**A**,**C**), respectively (see Methods). The apparent K_D_ values estimated for the above binding events are given in Table 1.

The availability of cation binding sites in the SF of the TOA^+^–KcsA complex has been explored by thermal denaturation experiments of such complexes in the presence of increasing concentrations of either permeant K^+^ or nonpermeant Na^+^. In these experiments, an excess TOA^+^ concentration of 100 µM, identical to that used in the crystallographic studies referenced above, was maintained constant throughout the titrations with the cations. Figure 2A,C shows representative binding curves for Na^+^, at both pH 7.0 and pH 4.0, respectively. Binding curves for Na^+^ to WT KcsA in the absence of TOA^+^ [37] are also included in all panels to facilitate comparison.

In these conditions, it is known that Na^+^ binds to a single set of sites provided by the S1 and S4 crystallographic sites in a nonconductive pore conformation [23] (PDB 2ITC). This binding process has a K_D_ in the millimolar range, slightly higher at pH 4.0 than at pH 7.0 (Table 1), which should correspond to the overall K_D_ for Na^+^ binding to the alluded S1 and S4 sites. In the TOA^+^–KcsA complex, access to the S4 site is blocked by the presence of TOA^+^; however, there is still Na^+^ binding to the available S1 site (Figure 2A,C), although the extent of thermal stabilization (on top of the thermal destabilization caused by TOA^+^) is lower and its K_D_ decreases an order of magnitude compared to the samples in the absence of TOA^+^ (Figure 2B,D and Table 1). These observations on Na^+^ binding to the available S1 site in the QA–KcsA complex are quite similar at pH 7.0 and pH 4.0 and analogous to the results previously seen in the presence of TBA^+^ [35,39].

**Figure 3:**
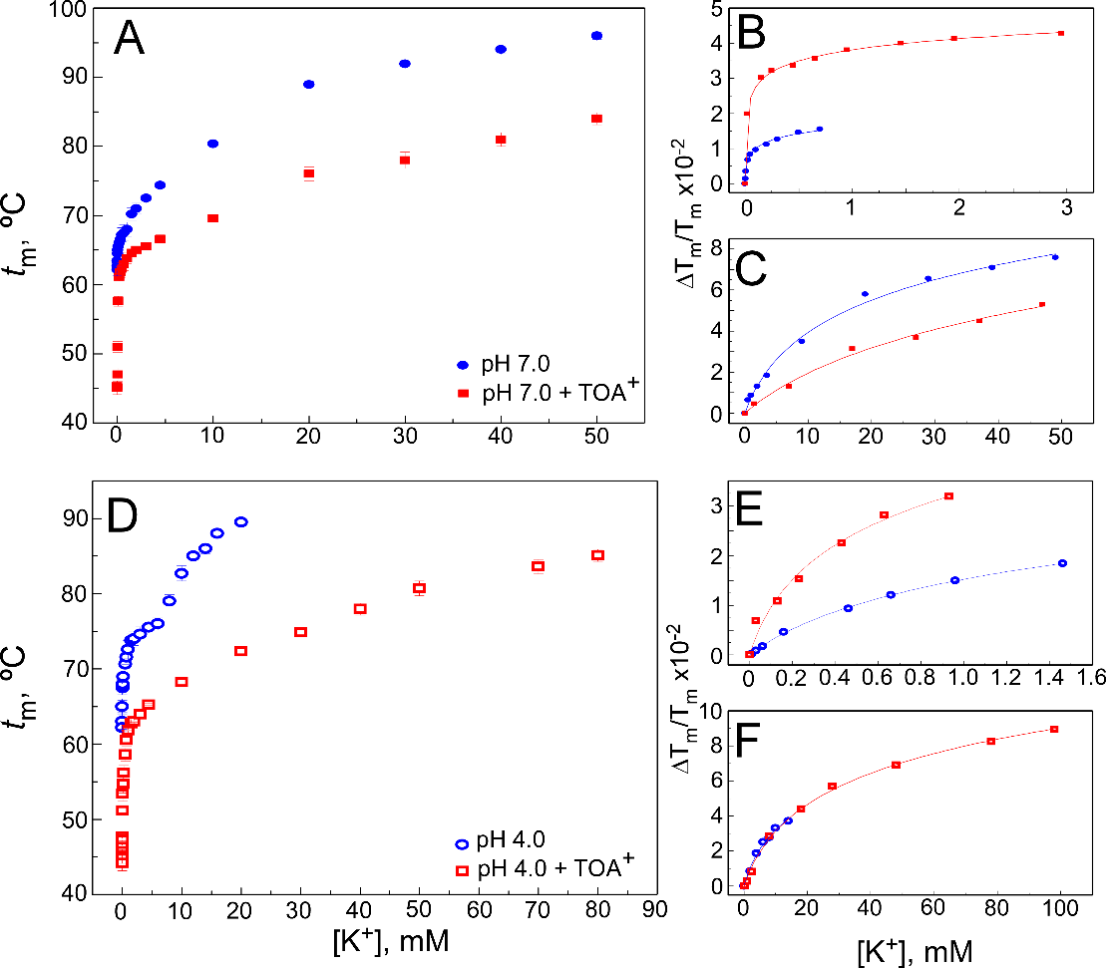
Effect of TOA^+^ on K^+^ binding to WT KcsA. Panels (**A**,**D**) illustrate K^+^ binding to the WT KcsA channel in the absence (circles) and presence of 100 µM TOA^+^ (squares) at pH 7.0 (panel (**A**)) or pH 4.0 (panel (**D**)), monitored through the K^+^-concentration-dependence of the midpoint temperature of the protein denaturation process (*t*_m_). Each experimental point is the average *t*m (in Celsius) ± S.D. (*n* = 3). When the low (panels (**B**,**E**)) and high (panels (**C**,**F**)) K^+^ concentration ranges are analyzed separately, Equation (1) adequately fits the data from panels (**A**,**D**), indicating that two different sets of K^+^ binding sites are present in the WT KcsA. The apparent K_D_ values estimated for the above binding events are given in Table 1.

Experiments similar to the above were also conducted at increasing K^+^ concentrations instead of Na^+^. As reported previously in the absence of QAs [37], K^+^ binds to two different sets of binding sites in WT KcsA at pH 7.0 (Figure 3A), which is consistent with crystallographic evidence on the ability of permeant cations to induce concentration-dependent transitions between nonconductive and conductive conformations of the SF [12,13]. The first set of such sites, assigned to the crystal S1 and S4 sites, shows a high affinity for K^+^ (micromolar K_D_), thus securing displacement of potentially competing nonpermeant cations. The second set of sites results from the contribution of all S1 to S4 crystallographic sites, is available only to permeant cations when the filter is in the conductive conformation and shows low affinity (millimolar K_D_) to favor cation dissociation and permeation. Figure 3B,C also shows that in the presence of a saturating concentration of TOA^+^, two different sets of sites are still available for K^+^ binding in the TOA^+^–KcsA complex, which, therefore, is clearly noncollapsed. This indicates that the TOA^+^-bound channel retains the ability to undergo K^+^ concentration-dependent transitions between different conformations of its SF. Despite such similarity, it is observed that the binding curve, mainly in the lower K^+^ concentration range, shows a lower slope than in the absence of TOA^+^, indicating a loss in K^+^ binding affinity. Indeed, Table 1 shows that the K_D_ values for the two K^+^ binding events in the TOA^+^–KcsA complex are increased with respect to those in the absence of the QA. This is particularly noticeable in the first, high affinity K^+^ binding event in which the K_D_ values differed by approximately two orders of magnitude. It should be noted that in the presence of TOA^+^, the entrance to the channel’s SF through the S4 site is permanently blocked by the bound TOA^+^. In the first K^+^ binding event, which takes place in the low K^+^ concentration range, the SF is in a nonconductive state, collapsed at the S2/S3 sites. This, along with the TOA^+^ blockade of the S4 site, indicates that the decreased affinity for K^+^ under the TOA^+^ blockade should be attributed to the binding of K^+^ to the S1 site in the nonconductive channel state. Nonetheless, as the K^+^ concentration increases, a second K^+^ binding event takes place, causing the filter to undergo a conformational transition in which the permeant cation reaches internal binding sites within the pore to provide the characteristic increase in thermal stability to the protein.

Figure 3D shows the results from experiments similar to those described in the previous paragraph but now conducted at pH 4.0 to induce channel inactivation. These experiments are further complicated because pH 4.0 causes an additional thermal stabilization of the channel protein compared to pH 7.0 [35]. Still, as described above for the resting channel at pH 7.0, both in the WT KcsA alone or when complexed to TOA^+^, the two sets of K^+^ binding sites are also detected in the K^+^ titrations of all samples at pH 4.0, when the channel is inactivated (Figure 3E,F). This was previously reported as a common feature in three different models of inactivated KcsA channels [35]. As expected for an inactivated state, all WT KcsA samples at pH 4.0 show a decrease in the affinity for K^+^. Indeed, Table 1 shows that when compared to the pH 7.0 samples, the K_D_s for K^+^ binding at pH 4.0 increase approximately three and two orders of magnitude, respectively, for the first and second K^+^ binding events. Such effects on the binding affinity caused by pH-induced channel inactivation are comparable to those caused by the presence of TOA^+^ bound to the resting state of the channel at pH 7.0. Interestingly, in contrast to TOA^+^, the shorter chain-length TBA^+^ does not critically change the interaction between the SF and the permeant cations [32,33,39].

### 2.3. The Homo-FRET Process in the W67 KcsA Mutant Reports Differences between the Nonconductive, Conductive, and Inactivated Conformations of the Selectivity Filter

**Figure 4:**
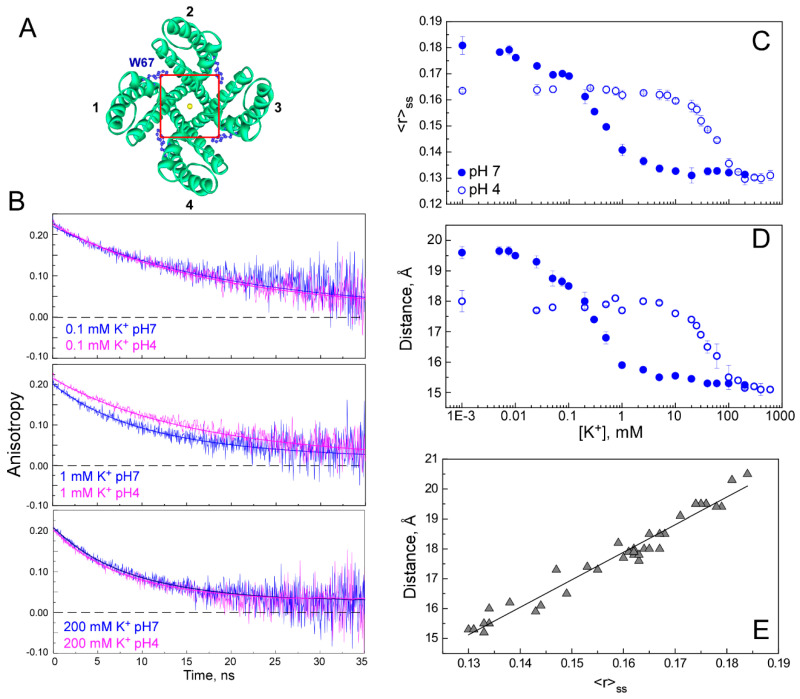
Influence of pH and K^+^ concentration on the homo-Förster resonance energy transfer (homo-FRET) process among the tryptophan residues in the W67 KcsA channel. Panel (**A**) shows a schematic top view of the tetrameric structure of KcsA (PDB: 1K4C; each monomer numbered 1 to 4) and the location of the W67 residues. Panel (**B**) illustrates representative fluorescence anisotropy decays obtained for W67 KcsA in the presence of 0.1, 1, and 200 mM K^+^ at both pH 7.0 (inner gate closed) and pH 4.0 (inner gate open). Equation (3) was then fitted to each data set, and the W67–W67 intersubunit distances were calculated from Equation (4). Panels (**C**,**D**) show the changes in the steady-state anisotropy <r>_SS_ (panel (**C**)) and in the W67–W67 intersubunit lateral distances calculated from the time-resolved anisotropy decays (panel (**D**)) at different K^+^ concentrations at both pH 7.0 and pH 4.0. Each titration curve is built from duplicate measurements from independent experiments (average ± S.D.) at each of the 20 to 25 different K^+^ concentrations covering the indicated concentration range. Panel (**E**) shows the linear correlation between the <r>_SS_ and W67–W67 lateral distances (distance = 92.165 × <r>_SS_ + 3.015; r^2^ = 0.960).

We recently reported an analytical framework to analyze the homo-Förster resonance energy transfer (homo-FRET) within a quadruple mutant W26, 68, 87, 113F KcsA channel bearing a single tryptophan residue (W67) per subunit (hereby called W67 KcsA) to characterize the interplay between the pore helix conformation and the cation occupancy at the SF [34] (Figure 4A). The ion channel activity of such W67 KcsA mutant was previously shown to be very similar to that of the WT KcsA channel [34].

The time-resolved anisotropy decays of this mutant protein at pH 7.0 (Figure 4B) show that the rate of the homo-FRET process (*k*_1_) increases in the presence of increasing K^+^ concentrations and, consequently, the steady-state anisotropy (<r>_SS_) and the W67–W67 intersubunit distance decrease along with it, further defining the characteristic two consecutive K^+^ binding events in the mutant channel (Figure 4C,D).

**Figure 5:**
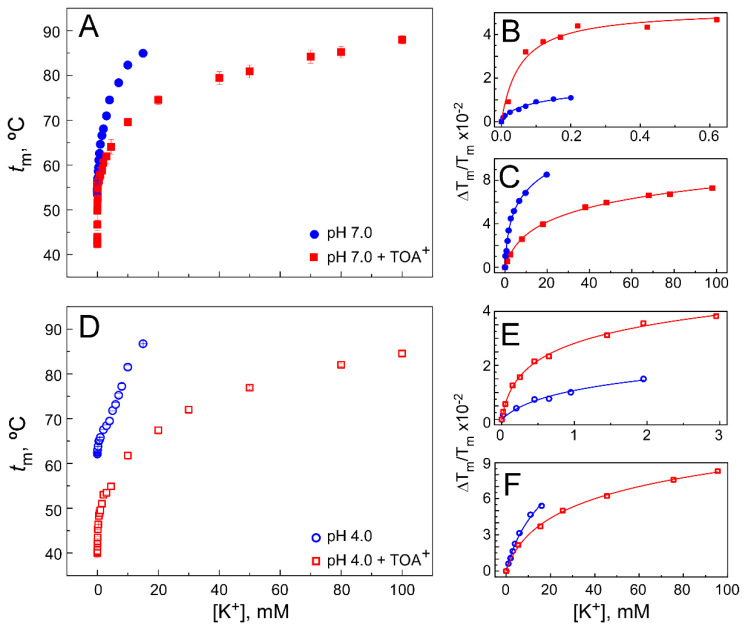
Effect of TOA^+^ on K^+^ binding to W67 KcsA. Panels (**A**,**D**) illustrate K^+^ binding to the W67 KcsA channel in the absence (circles) and presence of 100 µM TOA^+^ (squares) at pH 7.0 (panel (**A**)) or pH 4.0 (panel (**D**)), monitored through the K^+^-concentration-dependence of the midpoint temperature of the protein denaturation process (*t*_m_). Each experimental point is the average *t*_m_ (in Celsius) ± S.D. (*n* = 3). As to the WT KcsA from Figure 3, when the low (panels (**B**,**E**)) and high (panels (**C**,**F**)) K^+^ concentration ranges are analyzed separately, Equation (1) adequately fits the data from panels (**A**,**D**), which is evidence that the two different sets of K^+^ binding sites are present in W67 KcsA. The apparent K_D_ values estimated for the above binding events are given in Table 1.

Different from the samples at pH 7.0, we found that the first K^+^ binding event at K^+^ < 0.1 mM was no longer detected at pH 4.0, where the channel’s inner gate is open and the SF is inactivated. To test whether or not this first K^+^ binding event is present at pH 4.0, we ran complementary thermal denaturation experiments on the mutant W67 channel. In contrast to the observations from steady-state or time-resolved anisotropy fluorescence measurements, the thermal denaturation experiments showed that the W67 KcsA mutant behaves just like the WT-like channel as both K^+^ binding events were detected at either pH 7.0 or pH 4.0 (Figure 5).

Such apparent discrepancy between the two techniques could be simply explained by assuming that when the inner gate is opened by the acidic pH, the conformational changes involved in thermal stabilization at the low µM K^+^ range take place without significant modifications at the pore helix level, where the W67 reporter is located, as reflected by the maintenance of the steady-state anisotropy <r>_SS_ (~0.165) or the intersubunit distances (~18 Å), characteristics of the collapsed SF at low K^+^ concentrations and pH 7.0.

The comparison of anisotropy results from W67 KcsA at pH 7.0 and pH 4.0 at intermediate cation concentrations (the second K^+^ binding event) shows a clear decrease in K^+^ binding affinity in the inactivated, pH 4.0 state (Figure 4C,D). This is confirmed by parallel observations from thermal denaturation experiments using either WT or W67 KcsA channels (see Figure 3 and Figure 5 and Table 1). Additionally, when the K^+^ concentration is raised above 0.1 mM at pH 7.0 (channel in the closed-conductive conformation) or 5–10 mM at pH 4.0 (channel in the open-inactivated form), the <r>_SS_ and W67–W67 intersubunit distances decrease progressively to similar values of ~ 0.130 and 15 Å, respectively, in both cases. This indicates that the final conformations adopted by the SF at high K^+^ are indistinguishable in terms of intersubunit W67 distances (see Table 2), regardless of pH.

Interestingly, at both pH conditions, the K^+^-dependent changes in the steady-state anisotropy values and the W67–W67 intersubunit distances calculated from the time-resolved anisotropy decays (Figure 4C,D) show an excellent linear correlation. Therefore, the intersubunit distance for a given experimental condition can be easily estimated by simply interpolating the steady-state anisotropy value in the correlation curve shown in Figure 4E. Such a correlation curve was built with the data presented in this work, as well as data previously determined for saturating concentrations of different cations [34].

### 2.4. The Presence of TOA^+^ Bound at the Cavity Promotes the Inactivated State of the Selectivity Filter and Allosterically Changes the Pore Helix Conformation

Once the characterization of the K^+^ binding behavior of the W67 KcsA mutant channel at pH 7.0 and 4.0 was completed, we ran similar experiments but now in the presence of TOA^+^ in order to test the effect of this channel blocker on the SF and pore helix conformation and dynamics. Prior to that, control thermal denaturation assays showed that TOA^+^ binds to the W67 mutant channel in a manner similar to that observed when WT KcsA was used in the experiments. Indeed, the apparent dissociation constants and the corresponding confidence intervals for the TOA^+^–W67 KcsA complex, estimated at pH 7.0 and pH 4.0, were 10.7 (10.1–11.4) × 10^−8^ M and 5.2 (3.6–7.4) × 10^−8^ M, respectively. Furthermore, thermal denaturation assays of K^+^ binding to the W67 mutant channel in the presence of 100 µM TOA^+^ showed that binding of K^+^ to two different sets of sites was detected at either pH 7.0 or pH 4.0 (Figure 5). This is also similar to that previously observed in the WT protein and indicates that the stack of K^+^ binding sites at the SF remains available upon binding of TOA^+^ to the W67 mutant channel cavity.

**Figure 6:**
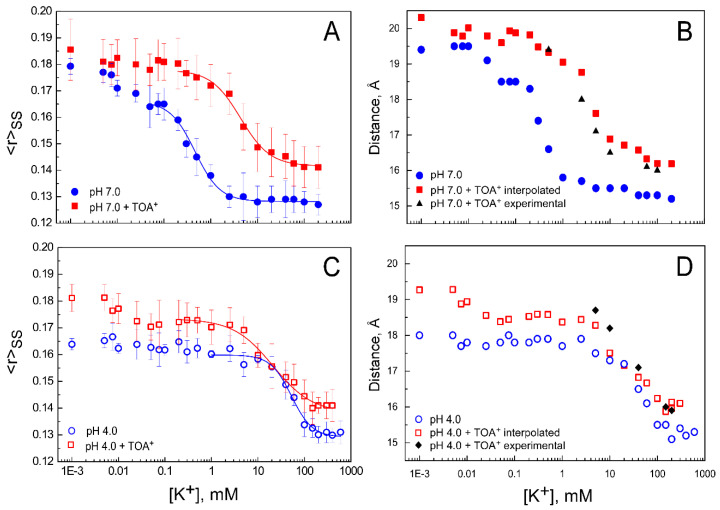
Effect of TOA^+^ on the conformation of the W67 KcsA channel. Panels (**A**) and (**C**) show a representative experiment of the dependence of the W67 <r>_SS_ on K^+^ concentration at both pH 7.0 and pH 4.0, in the absence or presence of 100 µM TOA^+^. The anisotropy values correspond to the average of ten measurements ± S.D. The solid lines represent the best fit of Equation (10) to the data. The apparent K_D_s for the above binding events were calculated from two independent experiments, and their average values, along with the confidence intervals, are given in Table 1. Panels (**B**,**D**) illustrate the dependence of the W67–W67 intersubunit distances on K^+^ concentration. The results from the samples without TOA^+^ were obtained experimentally from time-resolved anisotropy decays, as explained in Figure 4. In the samples containing 100 µM TOA^+^, such distances were obtained by simply interpolating the <r>_SS_ values using the correlation plot from Figure 4E. Nonetheless, the intersubunit distances in several samples containing 100 µM TOA^+^ were also determined from time-resolved anisotropy decays (black symbols) to test the goodness of the interpolation procedure.

In the homo-FRET experiments, two main effects of TOA^+^ binding to the W67 KcsA channel were detected: first, on the <r>_SS_ (and, thus, on the W67–W67 intersubunit distance) and, second, on the affinity of the SF to bind K^+^. As to the former, Figure 6A,C shows that the presence of TOA^+^ promotes an increase in the <r>_SS_ values at all the K^+^ concentrations tested compared to samples prepared in the absence of TOA^+^. Such an effect is much more noticeable at pH 7.0 than at pH 4.0 when the channel is already inactivated by the acidic pH. Indeed, the titration curve from the TOA^+^–containing samples at pH 7.0 closely resembles those obtained at pH 4.0, with or without TOA^+^, suggesting that similarly to the acidic pH, TOA^+^ by itself causes inactivation of the channel SF at pH 7.0. The <r>_SS_ values from above were interpolated from the correlation curve shown in Figure 4E to yield the corresponding W67–W67 intersubunit distances. As concluded from the <r>_SS_ values, it is observed that the intersubunit distances are increased in the TOA^+^–containing samples throughout the K^+^ titration curve and that such effects are more noticeable at pH 7.0 than at pH 4.0 (Figure 6B,D). This increase in the intersubunit distance at the SF and the resulting loosening of its structure seems consistent with the decreased thermal stability induced by TOA^+^ in these samples (Figure 3 and Figure 5). Finally, the intersubunit distances of several samples prepared in the presence of TOA^+^ were also determined experimentally from the time-resolved anisotropy decays. Such experimental values showed an excellent agreement with those calculated by interpolation from the steady-state anisotropy (black symbols in Figure 6B,D), attesting to the reliability of the simpler interpolation approach.

As to the effects of TOA^+^ on the affinity of K^+^ to bind to the SF, the titration curves in Figure 6A,B show that at pH 7.0, the sigmoidal decrease in <r>_SS_ or in the intersubunit distance in the TOA^+^-containing samples occurs at higher K^+^ concentrations than in its absence. This attests to a TOA^+^-induced loss in the affinity of the SF to bind K^+^. Again, this is not as noticeable in the samples at pH 4.0, in which the channel is already inactivated.

As indicated previously, the homo-FRET experiments monitor quite precisely the second K^+^ binding event to the KcsA channel, which corresponds to the K^+^-induced transitions from the nonconductive to either the conductive (pH 7.0) or the inactivated (pH 4.0) states. Therefore, in an attempt to quantitate the observed loss in affinity from the anisotropy changes, an analysis was performed by fitting the model described in Equation (10) (see Methods) to the data corresponding to the second K^+^ binding event in Figure 6A,C. At pH 7.0, the K_D_ value increases by an order of magnitude when TOA^+^ is present (0.45 to 4.3 mM), whereas at pH 4.0, it remains fairly constant (44 to 19 mM). Such K_D_ values derived from homo-FRET are in qualitative agreement with the K_D_ values estimated from thermal denaturation (Table 1 and Figure 5). Again, these observations further suggest that the binding of TOA^+^ causes inactivation of the channel SF at pH 7.0.

In addition to the main observations on the K_D_s for K^+^ binding, it was observed that the fit of Equation (10) to the data required a cooperativity parameter (*h*) higher than 1 only in the case of the pH 4.0 samples in the absence of TOA^+^ (a “*h*” parameter of 2, with a 95% confidence interval of 1.8 to 2.2, was used in those samples). This finding adds an additional feature to the pH-induced inactivated state of the channel since a cooperative binding process seems involved in facilitating the final inactivated conformation. A similar cooperative effect on the K^+^ binding to the open channel was previously described by NMR [21]. Such a presumed cooperative process, however, occurs at fairly high K^+^ concentrations and disappears when TOA^+^ is bound to the channel cavity.

## 3. Discussion

In this paper, a combined approach using thermal denaturation and homo-FRET assays was used to characterize the effects of TOA^+^ binding to KcsA. The addition of TOA^+^ to the channel protein induces a concentration-dependent decrease in protein thermal stability, opposite to the stabilizing effect observed of a shorter-chain QA, TBA^+^ [35,39]. When comparing the binding of these two QAs at pH 7.0 and pH 4.0, we observed that the closed-channel state at pH 7.0 exhibits a similar K_D_ for both TOA^+^ and TBA^+^. On the other hand, when the inner gate is open by pH 4.0, the affinity for TOA^+^ remains unaffected, while that for TBA^+^ decreases five orders of magnitude [35]. Since the only difference between these QA blockers is the length of their alkyl chains, it is concluded that the four extra carbon atoms in TOA^+^ are critical for better hydrophobic interaction with the protein channel wall. There is no crystallographic information on QA–KcsA complexes at pH 4.0, but based on related evidence [42,43,44], it seems reasonable to expect that the widening of the channel cavity accompanies the acidic-pH-induced untangling of the cytoplasmic α-helical bundle and the bending of the TM2 segment away from the symmetry axes of the channel. Such a widening of the cavity could diminish the hydrophobic component in the binding of the shorter TBA^+^, making it prompt to dissociate from the complex. However, the longer acyl chains of TOA^+^ extend further so as to traverse the channel protein wall completely [33]. This should keep it firmly associated with the hydrophobic residues even in the open conformation at pH 4.0, thus explaining why it maintains a high binding affinity. The X-ray crystallographic data at pH 7.0 also revealed that while TBA^+^ establishes van der Waals interactions with I100 and F103 residues from the cavity wall (TM2 helix), TOA^+^ adds interactions with L36 (from the TM1 helix), T74 (near the SF), and G99 and S102 (from the TM2 helix) [33]. These additional interactions between the protein and the QA should be involved in providing a higher affinity for the binding of TOA^+^ to the open state of the channel compared to TBA^+^, thus preventing its dissociation from the complex, as suggested by the earlier electrophysiological studies [5,6]. Another difference in the interaction between these two QAs with the channel consists of a change on the side-chain rotamer of the F103 residue in the TOA^+^–bound complex. The possible relevance of such observation is discussed below.

A thermal denaturation assay was also used to characterize the interaction of WT KcsA with Na^+^ and K^+^ in the presence of TOA^+^, in terms of both the number of binding events detected and their respective affinities. In the case of Na^+^, a single binding event with a slightly lower affinity than the control, in the absence of TOA^+^ (see Table 1), was observed, which corresponds to Na^+^ binding to its only available site, the extracellular S1 binding site. These observations on Na^+^ binding were quite similar at pH 7.0 and pH 4.0, or in the presence of TBA^+^, as indicated by the results. In contrast, the K^+^ binding studies indicated that the presence of bound TOA^+^ specifically affects the binding of the permeant species and is sensitive to the inner gate opening by the acidic pH. Thus, it is concluded that the effect of TOA^+^ on the ion–protein interactions specifically affects the binding to the channel of the permeant K^+^, and, therefore, monitoring of K^+^ binding becomes a useful tool to detect both the acidic pH-induced conformational change of the SF to an inactivated state and the changes induced by TOA^+^. As mentioned in the Introduction section, binding of K^+^ to WT KcsA is described by two consecutive binding events, with dissociation constants in the µM and mM range, respectively. Here, it is shown that in the presence of TOA^+^ bound to the channel cavity, the two K^+^ binding events still remain, suggesting that the SF, rather than collapsing, retains the ability to accommodate K^+^ at the stack of K^+^ binding sites and to undergo the K^+^-concentration-dependent conformational transition. Nonetheless, bound TOA^+^ induces a decrease in the affinity for K^+^ in both binding events at pH 7.0 when the inner gate is in the closed conformation. This is similar to that observed in the absence of TOA^+^ upon acidic-pH-induced channel inactivation. Therefore, this suggests that TOA^+^ binding by itself causes inactivation at pH 7.0 when the inner gate is closed. Indeed, a similar decrease in the affinity of the channel for K^+^ at pH 7.0 was also detected in mutant channels where the inactivation process is favored [35,45]. Furthermore, in apparent agreement with such conclusion, the presence of bound TOA^+^ in the pH 4.0 samples has only modest effects on the affinity of the two binding events for K^+^, likely because the channel is already inactivated. We have no evidence to propose a molecular mechanism to explain how TOA^+^ induces channel inactivation at neutral pH, but it could be speculated that the change on the side-chain rotation of the F103 residue in the TOA^+^–bound complex indicated above could be involved. The reason to speculate on such a possibility is that F103 is believed to be an essential residue in the allosteric crosstalk between the inner and outer channel gates [46,47,48] involved in the regulation of the channel’s functional cycle.

In order to gain structural information on the KcsA–TOA^+^–K^+^ complex, we use the quadruple mutant KcsA W26, 68, 87, 113F, which carries a single tryptophan (W67) as a fluorescent reporter of the SF conformation and dynamics. In this WT–like mutant channel, the homo-FRET process between the W67 residues from each subunit allows us to estimate the changes in steady-state anisotropy and the intersubunit lateral distances according to the type and concentration of cations within the SF [34]. Here, we first characterize in detail the acidic-pH-induced inactivated state in the absence of QAs. Even though the thermal denaturation assay from above detected two consecutive binding events for K^+^ at pH 4.0, the homo-FRET process is only sensitive to the transition from the nonconductive to the inactivated state. The analysis of this latter event shows a clear decrease in K^+^ binding affinity at intermediate concentrations of the cation, although the final conformation at saturating amounts of K^+^ is almost identical to that observed in the closed-conductive state.

As to the effects of TOA^+^ binding on the W67 mutant channel, we observed that TOA^+^ bound at the cavity allosterically modifies the conformation of the pore helices, leading to longer W67–W67 intersubunit distances at any K^+^ concentration at both pH 7.0 and pH 4.0. This loosening in the outer mouth packing seems consistent with the observed decrease in the thermal stability of the protein. The changes in the pore helix conformation, along with the decreased affinity for K^+^ at pH 7.0 caused by TOA^+^, seen in both homo-FRET and thermal denaturation experiments, are very similar to those effects caused by inactivation at pH 4.0. Therefore, as in the WT channel, it is concluded that TOA^+^ binding at pH 7.0 also causes channel inactivation in the W67 KcsA mutant.

The intersubunit distances determined from the time-resolved anisotropy decays can be compared to those calculated from the published X-ray data, which are usually obtained in the presence of Fab fragments bound to the channel to improve crystal resolution. Even though there are no X-ray data on WT KcsA at pH 4.0, some constitutively open mutant channels were successfully crystallized in different conditions [24,47,49]. Table 2 summarizes the W67–W67 distances calculated from the time-resolved anisotropy decays and compares them to the W67–W67 Cδ2-Cε2 lateral distances derived from X-ray data.

It is observed that the W67–W67 lateral distances calculated from the anisotropy decays of the open/inactivated W67 KcsA channel at pH 4.0 and high K^+^ concentrations (15.3 ± 0.1 Å) are almost identical to those W67–W67 Cδ2-Cε2 distances determine from X-ray data obtained in the presence of an intracellular Fab fragment (15.2 Å; PDB 3PJS), but not when an extracellular Fab was used to form the crystals (17.7 Å; PDB 3F5W). These results highlight how the binding of the extracellular Fab fragment alters the conformation of the extracellular loop and the SF dynamics. In fact, the same Fab fragment has been described to have a profound effect on KcsA inactivation [17]. In this respect, it should be noted that the available X-ray data on the TOA^+^–KcsA complex was obtained in the presence of the extracellular Fab fragment [33]. Based on such data, the authors concluded that the SF conformation in the TOA^+^–inactivated channel is a collapsed structure, similar to that detected in KcsA alone at low K^+^ concentrations [50] (PDB 1K4D), where the inner S2 and S3 K^+^ binding sites are absent. In contrast to such a conclusion, we find that the stack of K^+^ binding sites, although with a lower affinity, remains accessible in the TOA^+^–KcsA complex and that the W67–W67 intersubunit distances are very much like those found in the resting channel in the absence of TOA^+^. We attribute such discrepancies to the perturbing effects of the extracellular Fab fragment and/or to the C-terminal deletion on the X-ray data and conclude that rather than being collapsed, the inactivated TOA^+^–bound state of the SF at pH 7.0 and high K^+^ has a “resting-like” conformation. This is not unique to the inactivated TOA^+^–induced state as it is shared by several inactivated models of KcsA [35,51,52], and it seems, therefore, a general feature of the inactivated SF of KcsA.

In summary, the results obtained by the combination of the thermal denaturation assay and the analysis of the homo-FRET process among the W67 residues of each subunit in KcsA reinforce the argument that the long chain QA TOA^+^ stabilizes an inactivated conformation of the SF, as suggested by earlier electrophysiological studies [5,6], which is characterized by a lower affinity for K^+^ without affecting the interaction with Na^+^. However, in contrast to the conclusion from the X-ray studies, this inactivated state is not collapsed but, rather, in a “resting-like” conformation, where the differences between the conductive and inactivated SFs are more subtle. However, if the inactivated SF is “resting-like”, what makes it nonconductive? Earlier electrophysiological work concluded that inactivation is associated with a loss of K^+^ from the selectivity filter in potassium channels [53,54] and that the presence of the cations inside the selectivity filter is fundamental to stabilizing it in the conductive conformation [55,56,57]. In this respect, the drop in K^+^ affinity detected in our thermal denaturation and homo-FRET experiments would increase the probability of partial K^+^ depletion from the filter, thus hampering ion conduction.

## 4. Materials and Methods

### 4.1. Materials

N-Dodecyl-β-D-maltoside (DDM) ULTROL^®^ Grade was from Merck (Madrid, Spain). Hepes, succinic acid, N-methyl-D-glucamine (NMG), NaCl, KCl, tetraoctylammonium (TOA^+^) chloride, and dimethylsulfoxide (DMSO) were from Sigma-Aldrich (Madrid, Spain). Ni^2+^-Sepharose Fast Flow resin was from GE Healthcare (Madrid, Spain).

### 4.2. KcsA Heterologous Expression and Purification

The quadruple mutant of KcsA bearing a single Trp per monomer (W67 KcsA) was generated by mutating the rest of the native Trp residues 26, 68, 87, and 113 to Phe (W26, 68, 87, 113F). Wild-type (WT) and W67 channels were expressed in *E.coli* M15 (pRep4) and purified by Ni^2+^/His-tag affinity chromatography according to previous reports [34,58]. Proteins were purified in 20 mM HEPES buffer, pH 7.0, containing 5 mM DDM, 5 mM NMG, and different concentrations of KCl or NaCl. To prepare samples at pH 4.0, aliquots of the above were dialyzed against 10 mM succinic acid buffer, pH 4.0, containing 5 mM DDM, 5 mM NMG, and the corresponding amounts of NaCl or KCl. The tetrameric state of the protein was routinely checked by SDS-PAGE (12%) [59].

### 4.3. Thermal Denaturation Assay and Cation Binding Analysis

Thermal denaturation of WT and W67 KcsA channels was performed in a Varian Cary Eclipse spectrofluorometer by recording the temperature dependence of the protein intrinsic emission fluorescence at 340 nm after excitation at 280 nm, as previously described [37]. In these experiments, the protein was diluted to 1 µM protein concentration (in terms of monomers of KcsA) in either 20 mM Hepes buffer, pH 7.0, 5 mM DDM, and 10 mM NaCl (pH 7.0 buffer) or 10 mM succinic acid buffer, pH 4.0, 5 mM DDM, and 10 mM NaCl (pH 4.0 buffer), with additional amounts of NaCl, KCl, or TOA^+^, as required. The presence of 10 mM NaCl to start all titration experiments was required to maintain the tetrameric structure of the protein channels and to provide minimal stability to the proteins to start the thermal denaturation recordings.

When using TOA^+^, a concentrated stock (22 mM) was prepared in DMSO, and then aliquots from this stock were added to the samples and incubated for 30 min at room temperature. For titrations of either Na^+^ or K^+^ in the presence of TOA^+^, the same procedure as before was done, adding Na^+^ or K^+^ from a concentrated stock in the final step. The final amount of DMSO in the sample was always less than 1%, which, per se, has no effects on protein thermal stability.

The midpoint temperature of dissociation and unfolding of the tetramer (*t*_m_, in Celsius) was calculated from the thermal denaturation curves by fitting a two-state unfolding model to the data [38]. The dissociation constants of the KcsA–cation complexes (K_D_s) can be estimated from:

(1) $$\frac{\left|∆T_{m}\right|}{T_{m}}=\frac{\left|T_{m}-{\left(T_{m}\right)}_{0}\right|}{T_{m}}=\frac{R{\left(T_{m}\right)}_{0}}{∆H_{0}}\ln\left(1+\frac{\left[L\right]}{K_{D}}\right)$$

where T_m_ and (T_m_)_0_ refer to the denaturation temperature (in Kelvin) for the protein in the presence and absence of ligands, respectively, R is the gas constant, and ΔH_0_ is the enthalpy change upon protein denaturation in the absence of ligands. The change in T_m_ in the absence and presence of ligands (ΔT_m_) is expressed in absolute terms (|ΔT_m_|) since the presence of a given ligand can induce either an increase or a decrease of the observable value.

### 4.4. Steady-State Fluorescence Measurements

The steady-state fluorescence anisotropy of W67 KcsA was measured on a Horiba Jobin Yvon Fluorolog-3-21 or SLM 8000 spectrofluorometer and calculated as:

(2) $${<r>}_{\mathrm{SS}}=\frac{I_{\mathrm{VV}\;}-{G\cdot I}_{\mathrm{VH}}}{I_{\mathrm{VV}\;}+2{\;G\cdot I}_{\mathrm{VH}}}$$

where I_VV_ and I_VH_ are the fluorescence intensities (blank subtracted) of the vertically and horizontally polarized emission when the sample is excited with vertically polarized light, respectively, and the G factor (G = I_HV_/I_HH_) is the instrument correction factor. The samples were measured at 340 nm using an excitation wavelength of 300 nm [34]. A final protein concentration of 5 µM in either the pH 7.0 or pH 4.0 buffer, with or without TOA^+^, was used throughout. Ten measurements were done for each sample to calculate the average steady-state anisotropy values (±standard deviation).

### 4.5. Time-Resolved Fluorescence Measurements and Intersubunit Distance Calculations

Time-resolved fluorescence and anisotropy measurements with picosecond resolution were obtained using the time-correlated single-photon timing (SPT) technique. The fluorescence decays were measured at 345 nm (λ_exc_ = 300 nm) using an emission polarizer set at the magic angle (54.7°) relative to the vertically polarized excitation beam produced by a frequency-doubled Rhodamine 6 G laser [60].

The fluorescence and anisotropy decays were analyzed as described [34]. The W67–W67 intersubunit lateral distances were calculated from the time-resolved anisotropy measurements according to:

(3) $$r\left(t\right)=\frac{r\left(0\right)}{4}\;\left[1+e^{-4k_{1}t}+2e^{\left(-\frac{9}{4}k_{1}t\right)}\right]\cdot e^{-\frac{t}{\varphi_{g}}}$$

where r(0) is the initial anisotropy, *k*_1_ is the rate constant for homo-FRET between neighboring tryptophan residues, and φ_g_ is the rotational correlation time of the KcsA–DDM complex (43 ns) [34]. The intertryptophan lateral distance R can be directly calculated via *k*_1_:

(4) $$k_{1}=\frac{1}{\tau }{\left(\frac{R_{0}}{R}\right)}^{6}$$

where τ is the intensity-weighted mean fluorescence lifetime, and R_0_ is the critical radius computed with an orientation factor κ^2^ = 2/3. The goodness of fit was evaluated by statistical criteria (random distribution of weighted residuals and autocorrelation plots and a reduced χ^2^ < 1.2).

### 4.6. Calculation of the K^+^ Binding Affinity to KcsA from Changes in the Steady-State Anisotropy Values

The binding of K^+^ to KcsA W67, monitored by the changes in the <r>_SS_, presented a sigmoidal behavior that was also used to calculate the binding affinity of the channel for this permeant cation. Since we were mostly interested in the K^+^ binding event related to the equilibrium between the nonconductive (KcsA_(NC)_) and conductive (pH 7.0) or inactivated (pH 4.0) SF conformations of the channel (KcsA•K^+^_(C/I)_), we analyzed this second binding event, which can be described by a two-state equilibrium:

KcsA_(NC)_+ K^+^ ↔ KcsA•K^+^_(C/I)_

The total concentration of protein corresponds to:

(5) $${\left[\mathrm{KcsA}\right]}_{\mathrm{total}}={\left[\mathrm{KcsA}\right]}_{\mathrm{NC}}+{\left[\mathrm{KcsA}•K^{+}\right]}_{C/I}$$

and the dissociation constant (K_D_) is therefore defined by:

(6) $$K_{D}=\frac{{\left[\mathrm{KcsA}\right]}_{\mathrm{NC}}\cdot \left[K^{+}\right]}{{\left[\mathrm{KcsA}•K^{+}\right]}_{C/I}}$$

It could be assumed that the binding of a cation X^+^ to the protein is a cooperative process that is described by an empiric Hill function (sigmoid curve), so the free and bound molar fractions of KcsA as a function of cation concentration can be expressed as:

(7) $$x_{\mathrm{free}}=x_{\mathrm{NC}}=\frac{{\left[\mathrm{KcsA}\right]}_{\mathrm{NC}}}{{\left[\mathrm{KcsA}\right]}_{\mathrm{total}}}=\frac{{K_{d}}^{h}}{{K_{d}}^{h}+{\left[X^{+}\right]}^{h}\;}$$

(8) $$x_{\mathrm{bound}}=x_{C/I}=\frac{{\left[\mathrm{KcsA}\right]}_{C/I}}{{\left[\mathrm{KcsA}\right]}_{\mathrm{total}}}=\frac{{\left[X^{+}\right]}^{h}}{{K_{d}}^{h}+{\left[X^{+}\right]}^{h}\;}$$

where h is the Hill coefficient (cooperativity index).

The steady-state fluorescence anisotropy of a sample, <r>_SS_, prepared at a given X^+^ concentration is a weighted average of its limiting values, i.e., the steady-state fluorescence anisotropy of the nonconductive and conductive/inactivated conformations of KcsA, <r>_NC_, and <r>_C/I_, respectively. However, at variance with the usual case, the weighting factors here are the relative fluorescence intensities emitted by each species and not their molar fractions directly [61]. Mathematically, this is described as:

(9) $$<r>\;=\frac{x_{\mathrm{NC}}\cdot \Phi_{\mathrm{NC}}}{x_{\mathrm{NC}}\cdot \Phi_{\mathrm{NC}}+x_{C/I}\cdot \Phi_{C/I}}\cdot <r{>}_{\mathrm{NC}}+\frac{x_{C/I}\cdot \Phi_{C/I}}{x_{\mathrm{NC}}\cdot \Phi_{\mathrm{NC}}+x_{C/I}\cdot \Phi_{C/I}}\cdot <r{>}_{C/I}$$

where Φ_NC_ and Φ_C/I_ are the quantum yields of the nonconductive and conductive (pH 7.0) or inactivated states (pH 4.0), respectively.

If we define a parameter Q as the relative change in KcsA quantum yield upon K^+^ binding (Φ_C/I/_Φ_NC)_ and combine the equations from above, the general expression that can be used to fit <r>_SS_ data obtained in equilibrium binding studies is, therefore:

(10) $$<r>=\;<r{>}_{\mathrm{NC}}+\left(Q<r{>}_{C/I}-<r{>}_{\mathrm{NC}}\right)\cdot \frac{{\left[X^{+}\right]}^{h}}{{K_{d}}^{h}+{\left[X^{+}\right]}^{h}\;}$$

During the fitting procedures, the Q parameter was fixed at a constant value of 0.95 and 0.8 for the experiments carried out at pH 7.0 and pH 4.0, respectively.

## Figures and Tables

**Table 1:** Apparent dissociation constants (K_D_s) for the binding of Na^+^ and K^+^ to the WT KcsA channel at pH 7.0 and pH 4.0 in the absence or presence of 100 µM TOA^+^. Mean K_D_ values given here come from the experiments reported in Figure 2, Figure 3 and Figure 5. Since the estimated K_D_ values are derived from a logarithmic function (Equation (1)), we used the 95% confidence intervals of these values for statistical comparisons instead of giving mean ± S.D. values since only parametric analysis is appropriate on the logarithmic scale for such data. ^a^ Significant difference with respect to the same sample in the absence of TOA^+^ (*p* < 0.05). ^b^ Significant difference with respect to the same sample in the absence of TOA^+^ at pH 4.0 (*p* < 0.05).

Tested Cation	Sets of Binding Sites Detected	WT KcsA, pH 7.0				WT KcsA, pH 4.0			
		No Added TOA^+^		+100 µM TOA^+^		No Added TOA^+^		+100 µM TOA^+^	
		K_D_ (M) from Tm	95% CI	K_D_ (M) from Tm	95% CI	K_D_ (M) from Tm	95% CI	K_D_ (M) from Tm	95% CI
Na^+^	1	3.3 × 10^−3^	(2.5−4.3) × 10^−3 b^	1.4 × 10^−2^	(0.9–2.1) × 10^−2 a,b^	7.7 × 10^−3^	(6.6–8.9) × 10^−3^	1.3 × 10^−2^	(0.9–1.8) × 10^−2 a,b^
K^+^	2	2.8 × 10^−7^	(2.0–3.9) × 10^−7 b^	5.0 × 10^−5^	(3.3–7.5) × 10^−5 a,b^	3.8 × 10^−4^	(2.1–6.9) × 10^−4^	1.9 × 10^−4^	(0.9–4.9) × 10^−4^
		4.2 × 10^−3^	(3.2–5.4) × 10^−3 b^	1.6 × 10^−3^	(1.2–2.1) × 10^−2 a,b^	1.1 × 10^−1^	(0.7–1.5) × 10^−1^	1.0 × 10^−1^	(0.8−1.4) × 10^−1^
		**W67 KcsA, pH 7.0**				**W67 KcsA, pH 4.0**			
		**K_D_(M) from Tm**	**95% CI**	**K_D_(M) from Tm**	**95% CI**	**K_D_(M) from Tm**	**95% CI**	**K_D_(M) from Tm**	**95% CI**
K^+^	2	1.0 × 10^−5^	(0.7–1.5) × 10^−5 b^	3.2 × 10^−5^	(1.2–8.6) × 10^−5 b^	4.8 × 10^−4^	(2.7–8.7) × 10^−4^	1.2 × 10^−4^	(1.0−1.4) × 10^−4 a,b^
		9.4 × 10^−4^	(5.8–14.0) × 10^−4 b^	5.2 × 10^−3^	(3.3–8.1) × 10^−3 a,b^	1.4 × 10^−2^	(1.1–1.7) × 10^−2^	8.6 × 10^−3^	(7.1–10.4) × 10^−3 a,b^
		**K_D_(M) from <r>_SS_**	**95% CI**	**K_D_(M) from <r>_SS_**	**95% CI**	**K_D_(M) from <r>_SS_**	**95% CI**	**K_D_(M) from <r>_SS_**	**95% CI**
K^+^	1	4.5 × 10^−4^	(3.6–5.4) × 10^−4 b^	4.3 × 10^−3^	(3.4–5.2) × 10^−3 a,b^	4.4 × 10^−2^	(4.3–4.6) × 10^−2^	1.9 × 10^−2^	(1.2–2.5) × 10^−2 a,b^

**Table 2:** Comparison of the W67–W67 intersubunit distances calculated from the available X-ray crystallography data and from the homo-FRET analysis of the W67 KcsA mutant channel.

Inner Gate Conformation	Ionic Condition	*Intersubunit Distances* (Å)		
		*C(δ2)-C(ε2) Inter-Tryptophan Lateral Distances* from X-ray Structures ^(a)^		Homo-FRET Analysis ^(b)^
		Extracellular Fab, 1–125 KcsA	Intracellular Fab, full-length KcsA	
Closed	Low K^+^	17.8 (1K4D)	n.a. ^(c)^	18.5 ± 0.2
	High K^+^	17.3 (1K4C)	15.9 (3EFF)	15.4 ± 0.2
	Low K^+^ + TOA^+^	n.a.	n.a.	19.1 ± 0.3
	High K^+^ + TOA^+^	17.6 (2W0F)	n.a.	16.1 ± 0.2
Open	Low K^+^	n.a.	n.a.	17.9 ± 0.2 ^(e)^
	High K^+^	17.7 ^(d)^ (3F5W)	15.2 ^(d)^ (3PJS)	15.3 ± 0.1 ^(e)^
	Low K^+^ + TOA^+^	n.a.	n.a.	18.4 ± 0.2 ^(e)^
	High K^+^ + TOA^+^	n.a.	n.a.	16.2 ± 0.3 ^(e)^

(a) PDB accession numbers of each crystallographic structure are given in parentheses. (b) The calculated distances from the homo-FRET analysis in the W67 mutant channel represent the mean ± S.D. of at least two independent experiments performed at pH 7 (closed state) or pH 4.0 (open state). (c) n.a., not available. (d) The X-ray data from the open states were collected at pH 7.0 using constitutively open mutant channels. (e) The opening of the inner gate was induced by acidic pH.

## Data Availability

Data is contained in the article.
